# “A very good program … but I still have the knee problem”: A qualitative study exploring patient acceptability of physiotherapy-led osteoarthritis services

**DOI:** 10.1016/j.ocarto.2026.100799

**Published:** 2026-05-05

**Authors:** Alison J. Gibbs, Allison M. Ezzat, Jason A. Wallis, Joanne L. Kemp, Nicholas F. Taylor, Juanita Low, Christian J. Barton

**Affiliations:** a La Trobe Sports and Exercise Medicine Research Centre, La Trobe University, Bundoora, Australia; b School of Allied Health, Human Services and Sport, La Trobe University, Bundoora, Australia; c Physiotherapy Department, Eastern Health, Box Hill Hospital, Melbourne, Australia; d Department of Physical Therapy, Faculty of Medicine, University of British Columbia, Vancouver, BC, Canada; e School of Public Health & Preventive Medicine, Monash University, Melbourne, Australia; f Allied Health Clinical Research Office, Eastern Health, Box Hill, Australia

**Keywords:** Knee osteoarthritis, Qualitative research, Physical therapy, Community health services

## Abstract

**Objective:**

What is the acceptability of community-based physiotherapy-led osteoarthritis services, including advanced practice assessment and an exercise and education program, for patients with knee osteoarthritis?

**Design:**

Interpretive descriptive qualitative study. Patients with knee osteoarthritis who attended a community-based advanced practice physiotherapy assessment, followed by physiotherapy management, including an osteoarthritis-specific education and exercise program (Good Life osteoArthritis:Denmark (GLA:D®)) were eligible. Semi-structured interviews, informed by the Theoretical Framework of Acceptability, were completed either online or via telephone. Interview data were independently coded by 2 researchers. Other team members reviewed a random sample of interview data. Data were analysed inductively using thematic analysis.

**Results:**

Twenty participants (mean 70 years, 60% women) were included. Three main themes were identified: *1. Physiotherapy-led osteoarthritis services were acceptable to most participants; 2. Physiotherapy care received was typically perceived as insufficient to fully addressallongoingknee osteoarthritismanagement needs* and *3. Effective clinical and non-clinical communication was strongly linked to acceptability of physiotherapy-led osteoarthritis services.*

**Conclusion:**

Assessment by community-based advanced practice physiotherapists and participating in a physiotherapy-led exercise-and-education-program were acceptable to most patients, providing a viable alternative to hospital-based models. However, several barriers were identified, including patient uncertainty regarding addressing their education needs and ongoing management needs. Prioritising ongoing management, communication and a strong therapeutic alliance can enhance acceptability. Our findings highlight challenges and enablers for implementing physiotherapy-led care in a community-based setting, and may guide implementation of these services in alternative healthcare settings.

## 1 Introduction

Higher quality guidelines concur that exercise, education and weight loss (if required) are first-line care and recommended for all people with knee osteoarthritis [1]. Surgery should only be considered after receiving appropriate non-surgical management, centred on first-line care [1]. However, most people do not adequately access first-line care [2,3], and general practitioners are three times more likely to refer to orthopaedic surgeons than physiotherapists [2]. Reported barriers to first-line care include health professional beliefs being inconsistent with guidelines, a perceived lack of effective first-line care service options, time constraints, and high cost [[4], [5], [6]]. People with osteoarthritis report lacking desire to exercise, difficulty accessing services and challenges navigating healthcare [4,6,7].

Physiotherapist-led models of care for managing osteoarthritis have been implemented in Australia and internationally [8], including advanced practice physiotherapist services assessing people with osteoarthritis referred for orthopaedic specialist opinion [9,10]. These services arrange non-surgical management where indicated, including consideration of first-line care [9], and refer appropriate patients for surgical opinion when indicated [11]. Advanced practice physiotherapists make similar decisions to orthopaedic consultants [9], assist in reducing orthopaedic waiting lists [11], and improve health system efficiency [11]. Australian advanced practice physiotherapists typically have post graduate qualifications, extensive clinical experience, and undergo a credentialling process guided by a national competency framework [12].

More than 60% of people accessing Australian advanced practice physiotherapy clinics for knee osteoarthritis are referred for non-surgical management [13,14]. This commonly includes referral to physiotherapist-led osteoarthritis education and exercise programs, which can improve pain and function, and reduce desire for surgery [15,16]. However, like orthopedic surgeon waitlists, long waits for hospital-based advanced practice physiotherapy clinics are reported [14]. Additionally, only 60% of those referred to physiotherapy actually attend [17]. Providing an advanced practice physiotherapy service in a community health service instead of a hospital is feasible [14], and with provision of onsite osteoarthritis programs, may facilitate greater uptake of first-line care.

Patient acceptability is vital for an intervention to be successful [18]. Acceptability can be influenced by attitudes to potential care, including appropriateness, suitability and perceived effectiveness, as well as satisfaction with care provided and outcomes [19]. Patient satisfaction with advanced practice physiotherapist roles has been reported to be either equal or superior to care provided by orthopaedic doctors [11,20]. Additionally, both community-based and hospital-based advanced practice osteoarthritis services achieve similarly high patient satisfaction [14]. However, these previous evaluations of satisfaction [11,14,20] are mostly limited to quantitative questionnaire-based outcomes and may not comprehensively capture all aspects of acceptability [21]. A more comprehensive understanding of the acceptability of community-based physiotherapy-led osteoarthritis services, via qualitative interviews, may assist in developing community-based advanced practice services and strategies to increase the uptake of non-surgical management.

Therefore, the research question was: What is the acceptability of Australian community-based physiotherapy-led osteoarthritis services, including advanced practice assessment and an exercise and education program, for patients with knee osteoarthritis?

## 2 Method

### 2.1 Design

This qualitative study used semi-structured interviews and is reported in line with COnsolidated criteria for REporting Qualitative studies (COREQ) [22]. Our qualitative approach was interpretive description, a non-categorical approach allowing incorporation of researchers knowledge and experience, aiming for understanding of phenomena to develop clinical implications, rather than develop theory [23]. St Vincent's Public Hospital Melbourne (HREC 251/21) provided ethics approval.

### 2.2 Participants

Eligible participants were patients able to communicate in English who were participating in the pre-registered cohort implementation study MonitOring The Influence Of care in people with kNee osteoarthritis (MOTION) [24], and who had attended an advanced practice physiotherapy assessment at one of three local community health services. Details about the MOTION study are reported separately [25]. Briefly, it involves a large cohort study of people with knee osteoarthritis referred to Victorian public hospitals, with a subgroup given support to access physiotherapist-led first-line care in a community health service. Community health services are independent, not-for profit organisations offering partially government subsidised allied health services [26]. Participants in the MOTION study received timely access to community-based advanced practice physiotherapy assessment and triage, and fully subsidised out-of-pocket costs to attend physiotherapy (including Good Life osteoArthritis: Denmark (GLA:D®), a physiotherapy-led, group-based education and exercise program for hip and knee osteoarthritis [15]) and dietetics. Participants unable to attend GLA:D® at the community health service could attend GLA:D at a private practice (either in person or via telehealth), with fees fully subsidised. Exclusion criteria included patients categorised for urgent orthopaedic appointment, knee osteoarthritis not the primary complaint, GLA:D® completion within the last 12 months, rheumatological conditions, neurological conditions, cognitive impairments affecting ability to engage in structured education and exercise, medical advice not to engage in exercise, and previous osteotomy or metal implants at the relevant knee [25]. Eligible participants for this study were contacted by telephone after completing their physiotherapy care, and provided with verbal information about the study. All study participants provided written informed consent, returned via pre-paid post.

For this study, purposive sampling was used to ensure participants from all three partnering community health sites, and variation in willingness for surgery at baseline and follow up – determined by asking participants “If you could have knee joint replacement surgery within the next week and it was a convenient time for you, would you undergo the procedure?”, with answer options “yes”, “unsure” and “no”.

### 2.3 Data collection

Demographic data (age, indigenous status, employment status, Knee Osteoarthritis and injury Outcome Score (KOOS) joint-related quality of life subscale [27], pain numerical rating [28], body mass index (BMI), surgical willingness, and radiological severity of osteoarthritis (Kellgren-Lawrence Score) [29]) were collected as part of the MOTION study. Semi-structured interviews were conducted online (Zoom) or by telephone, based on participant preference, by two interviewers (JL and AE) using a topic guide developed by the research team and informed by the Theoretical Framework of Acceptability (Appendix 1) [19]. The topic guide was not piloted, but was refined iteratively over consecutive interviews. Interviews were audio recorded and transcribed verbatim by a professional transcription service (Transcription Australia, www.transcription.net.au).

The interviewers were female physiotherapists: one a researcher with 20 years clinical experience managing knee osteoarthritis and extensive qualitative experience (AME), and one a PhD candidate (JL), who received qualitative training and support from experienced researchers (CJB and AME). The interviewers understood the advanced practice service and the GLAD® program, but were not directly involved in providing care. Interviews were reviewed iteratively by the research team. Once the research team determined no new ideas were being identified, a further two interviews were conducted to confirm sufficient information power had been achieved [30].

### 2.4 Data analysis

Demographic data were reported descriptively. All analysis team members were registered physiotherapists, two were PhD candidates (AJG and JL) and the remaining were experienced researchers (range 6–24 years post PhD). Researcher AJG has training and experience in qualitative research, and researchers CJB, AME, JAW and NFT have extensive qualitative research experience. One researcher (AJG) worked as an advanced practice physiotherapist in public health and at one of the recruitment sites. She was cognisant of potential influences of her background on data interpretation, but this also allowed for in-depth understanding of the role and setting. She was not involved in coding interviews from that site initially. After three other research team members reviewed the data from that site, she reviewed the transcripts, facilitating indepth understanding of all the data.

Field notes were documented after each interview and reviewed by the team. Five researchers (AJG, CJB, JLK, JAW and AME) were involved with training clinicians to deliver GLA:D®. To provide theoretical triangulation and multiple perspectives, all research team members provided feedback on the codes and themes.

All transcripts were reviewed by AJG for transcription accuracy. We applied a six step inductive thematic analysis approach to identify themes [31]. Transcripts were read through iteratively by researcher AJG. Initial descriptive codes based on a sub-set of interviews (n = 9) were developed inductively by AJG, with data organised using NVivo 14 software. Researchers AME and CJB listened to recordings to allow familiarisation with the data, and provided reflective feedback on AJG's codes and interpretations. The remaining interviews were then inductively coded independently by AJG, with AME and CJB both independently coding 50% of the interviews. Codes were then reflected on and discussed by researchers AJG, CJB and AME to ensure alternative perspectives were considered. Additionally, researchers JLK, NFT and JAW read a sample of 50% of the interview transcripts. All research team members met once to review, define and name initial themes. Themes were further refined by the wider research team through sharing, reflecting on and feeding back on five text document versions.

## 3 Results

Thirty-eight participants were invited for interview, three declined, ten were non-English speaking, three did not return a consent form, and one was uncontactable after returning the consent form. Twenty-one participants consented and were interviewed. One participant was excluded after interview upon discovering they had attended a hospital-based advanced practice service, and not a community health service. Thus, 20 interview transcripts were included, exceeding the 9–17 previously reported as an appropriate sample size for qualitative research [32]. Twelve (60%) were females, mean (SD) age was 70 (10) years, mean (SD) BMI was 29.3 (4.8), and 15 (75%) were willing for surgery or perceived surgery would be required (Table 1 and Appendix 2). Characteristics for interviewees were similar to the wider MOTION cohort (Table 1). Most (n = 17, 85%) attended 10 or more group exercise sessions and one or more education sessions (Table 2 and Appendix 2). Thirteen (65%) were either willing for surgery or perceived surgery would be required at 4 months (Appendix 3). The average interview duration was 32.2 (range 13.5–56.0) minutes.

**Table 1:** Baseline characteristics.

Variable	Interviewees n = 20	MOTION Cohort n = 318
Age, mean years (SD)	70 (10)	67 (10)
Women, percentage, (number)	60 (12)	57 (182)
BMI kg/m2 mean (SD)	29.3 (4.8)	32.2 (7.3)
Work status, percentage (number)		
Unemployed	0 (0)	3 (11)
Retired	55 (11)	53 (169)
Not working due to knee	10 (2)	6 (18)
Not working other reasons	5 (1)	5 (15)
Working reduced hours due to knee	0 (0)	2 (5)
Working part-time	15 (3)	14 (45)
Working full time	15 (3)	17 (55)
Born in Australia, percentage (number)	75 (15)	65 (206)
English as a first language, percentage (number)	85 (17)	84 (267)
Highest educational level attained, percentage (number)		
Did not complete primary school	0 (0)	3 (9)
Primary school	10 (2)	20 (63)
High school	10 (2)	18 (56)
Apprenticeship	5 (1)	8 (26)
Certificate	10 (2)	18 (57)
Diploma	15 (3)	15 (47)
Undergraduate degree	15 (3)	9 (30)
Postgraduate degree	35 (7)	9 (30)
Identifies as Aboriginal or Torres Strait Islander, percentage (number)	0 (0)	1 (4)
KOOS subscale knee-related QOL mean (SD)	40 (14)	30 (19)
Worst pain NRS, mean (SD)	69 (22)	74 (22)
Willingness for joint replacement now^a^, percentage (number)		
Yes	45 (9)	61 (194)
Unsure	10 (2)	23 (74)
No	45 (9)	16 (50)
Need for future joint replacement surgery^b^, percentage^c^ (number)		
Yes	55 (6)	71 (88)
No	45 (5)	29 (36)
Total perceiving surgery required percentage (number)	75 (15)	89 (282)
KL score, percentage (number)		
Grade ≤2	30 (6)	23 (74)
Grade 3	40 (8)	44 (141)
Grade 4	25 (5)	25 (79)
MRI only	5 (1)	5 (17)
Not available	N/A	2 (7)

BMI=Body Mass Index; KL=Kellgren Lawrence; KOOS = Knee Outcome Osteoarthritis Subscale; NRS= Numerical Rating Scale; QOL = Quality of Life; a = Willingness to undergo Total Knee Arthroplasty within the next week; b = Response to question “Do you think you will need knee replacement surgery at any time in the future?” c = percentage of those who responded no or unsure to surgical willingness now question.

**Table 2:** Summary of care attended.

Care attended	Participants
Completed 10 or more GLA:D® exercise sessions, percentage (number)	85 (17)
Attended 1 or more education sessions, percentage (number)	85 (17)
Attended 2 or more 1:1 physiotherapy sessions, percentage (number)	35 (7)
Attended GLA:D® at community health service, percentage (number)	85 (17)
Attended GLA:D® at private practice, percentage (number)	15 (3)

GLA:D® = Good Life osteoArthritis: Denmark.

Three main themes (and seven subthemes) were identified: *1. Physiotherapy-led osteoarthritis services were acceptable to most participants; 2. Physiotherapy-led osteoarthritis care received was typically perceived as insufficient to fully address* *all* *ongoing* *knee osteoarthritis* *management needs* and *3. Effective clinical and non-clinical communication was strongly linked to acceptability of* *physiotherapy-led* *osteoarthritis services.* Themes 1 and 2 had supportive subthemes. Illustrative quotes for all themes are provided in text and a larger sample of quotes presented in Table 3 and Appendix 4.

**Table 3:** Themes and subthemes with illustrative quotes.

Theme/subthemes	Supportive quotes
***Theme 1. Physiotherapy-led osteoarthritis services were acceptable to most participants*** An advanced practice assessment was acceptable to most participants, but often not strongly desired.	• *And, as I say, I felt entirely confident XX knew what they were doing* • *[The advanced practice physiotherapist] is more specialised in, I think, for knee problem … [The advanced practice physiotherapist] very thorough … really is wonderful. They are so patient and so experienced (ID#15)* • *Yes, I felt much more comfortable with that [seeing advanced practice physiotherapist instead of surgeon] (ID#20)* • *Great [to see advanced practice physiotherapist instead of surgeon] ‘cause the last thing I wanna see is a surgeon. There's no doubt – I just don't wanna have to have the surgery if I don't need to [ID#8]* • *I think really it felt as a formality. It wasn't really anything much more … I didn't really understand quite why I had to see [the advanced practice physiotherapist] … I felt it was for them rather than me (ID#16)* • *It all seemed a bit superficial and a bit – I walked away going, “I wasn't asked questions that I would have liked to be asked” (ID#6)* • *I would like to have gone straight to an orthopaedic person because they know everything about the knee and seen and heard it all (ID#1)* • *I wasn't overly worried or anything [about seeing an advanced practice physiotherapist] because I was told that if I wanted to have the knee replacement, I could at any time and I just had to let them know (ID# 9)* • *Yes, I would have preferred to see a doctor or surgeon and then he could have said “Better you do this physio and we'll take it from there.” (ID#12)* • *I was pleased to go local instead and go relatively quickly as against waiting to see somebody in months and months and months (ID#1)*
Exercise is perceived as beneficial and acceptable by most participants, including supervision from a physiotherapist and being in a group environment, but not often strongly desired	• *Yeah. I'm quite pleased with my progress and walking around without much pain is very good. (ID#9)* • *And I did well and they [the exercises] worked for me, like it lessened my feeling of weakness in the knee (ID#5)* • *I think I did the full gamut of them and I found them – well, I quite enjoyed doing it really. I'm not sure how much it did for me, but it alerted – you know, it was a regime that put it into your mind, well, you know, just think about things. (ID#7)* • *Strengthen the knees, strengthen the leg, the muscle around it, so that you can manage the pain (ID#15)* • *Well, it was very helpful. The pain in my knee went and I was able to walk more freely without the limp, and I was able to do more things around the house and that … thanks to the exercises. (ID#9)* • *The GLA:D program has been brilliant. (ID#8)* • *I was a bit disappointed … I didn't have the better results I thought I would have (ID#6)* • *I don't know where it went wrong. At some session, I just had some discomfort. (ID#13)* • *The very first time when you go, it makes you feel like you're not the only one (ID#10)* • *You're doing it [at home] more because “oh, no, I really should be doing it”. Whereas, when you go down [to the group], you want to do it because you want to get better and you're around people who have got that same feeling (ID#10)*
Participants views on education sessions were mixed	• *It's just there was a lot of information that I found interesting … I would say they offered a lot of benefit, actually (ID#11)* • *The way I was able to ask questions, what their presentation was and all that, not only clarified it but it made me feel better about it (ID#9)* • *I can't really remember what they say. I don't remember, like, in a few weeks, I think it's been months now. (ID#13)* • *The physio couldn't answer any of our questions. And basically, the education class was such a waste that we could have just done it by reading the paper. That's all [the physiotherapist] did. [They] just read out the handout (ID#6)*
***Theme 2. Physiotherapy care received was typically perceived as insufficient to fully address all ongoing knee osteoarthritis management needs*** Exercise is not perceived as a long-term solution	• *The evidence from whatever it was, a CT scan or something, whatever kind of scanning and X-raying, that was actually showing that my cartilage in my right knee had basically gone … I mean there was a clear imaging correlation between the way I was feeling and the pain I was feeling (ID#16)* • *My knee problem is more of a structural thing that can't be fixed totally by exercise (ID#4)* • *Exercise [has improved knee] Less pain, better movement, sleeping better – just generally improvement. It's still sore, yeah it's never gonna be the same. They can't put a cartilage back in there (ID#8)* • *It [exercise] didn't cure anything (ID#5)*
Ongoing support (both exercise group and monitoring) was commonly desired by participants	• *If they could offer physio – I don't know – once a month or once every five weeks just to see that it isn't getting critical (ID#01)* • *I just think if they had the program goes on a little bit longer, the exercise part of it would be beneficial, I think, to a lot of people (ID#20)* • *I think if the initial program was 12 weeks rather than – I think I did ten, it was ten, then, yeah, that could only be good. I mean one would be that much fitter. The real challenge is what happens afterwards (ID#16)* • *You're out there on your own. And I've got no one that I can ring up and say, look, you know, some sort of reassurance (ID#12)*
Uncertainty regarding future options and prognosis	• *I just don't know what the future holds for my knee. I don't know how bad it will get. It's really hard to say. And the trouble is if it gets so bad that I say, “Yes, I want one [joint replacement]* *,” I've got maybe a year's wait … You're juggling a whole lot of ‘what if’ and likelihoods (ID#06)* • *Well, if there are changes to it, I need to understand are they better or are they worse? If they're better, then as far as GLA:D is concerned, is it something that I can continue through [the health service] or GLA:D, or do I have to start doing exercises somewhere else on my own?* *If it is worse, what does [the advanced practice physiotherapist] recommend? Where should I – what is my next step for this, ones that I should consider? (ID#10)*
Surgery was often perceived as inevitable and the optimal treatment, although often a last resort	• *I'm holding that [surgery] out as going to be the problem solved once I have a knee reconstruction (sic) [replacement] (ID#01)* • *There's times, where it's pretty tough, so I do need the surgery there on that, for sure (ID#18)* • *Well, I can't help feeling that if something was fixed by surgery (ID#04)* • *I still surf, but I just do it a different way. So, I'm happy with that (ID#05)* • *I've known people where surgery hasn't worked. It's more difficult for them in the future [after surgery]. And I've known other people that it's worked great. So I would rather, if I can, avoid going for it and still be able to walk and move (ID#14)* • *I think it will get to a point where it will have to be done, but it's at what point that is. Like, is it* *12 months' time or is it four years' time? (ID#11)* • *I'm not really keen to have that done, because, as I said, I've got lymphedema in the lower part of my legs … I just don't wanna risk any infection and then lose half a leg or something (ID#20)* • *If you'd asked me that* *12 months* *ago, I would have jumped at it … just I'm confident now the program has got me to a stage where I'm not going to need surgery. If I stop, I think it'll deteriorate, but I'm just going – just got to keep going with the exercises. (ID#08)*
***Theme 3: Effective clinical and non-clinical communication was strongly linked to acceptability of physiotherapy-led osteoarthritis services***	• *I can't speak highly enough of the positivity, the confidence, the manner, the respect I was given (ID#02)* • *The communication was very good. The questions were simple and straightforward. I felt that [the advanced practice physiotherapist] was answering the questions (ID#13)* • *And I always felt someone was on the end of the phone easily should I need to query anything (ID#02)* • *It was a bit odd, they were a bit vague and a bit – they didn't strike me as being terribly professional or terribly knowledgeable, which I thought was quite strange … I just was a bit surprised by the casualness of it in the – not really taking it terribly seriously, I felt (ID#06)* • *And I think [the advanced practice physiotherapist] said [they'd] see me again in December, but I haven't heard from [them] and it's December now. So, I don't know what's happening there … I wouldn't say disappointing but confusing anyway (ID#03)* • *But [XX community health service], I'll tell you something, they ring you up, they ring four times. And sometimes you're driving, you can't answer. And when you try to ring them back, it is murder. One day, it was Friday, I rang seven times, and they put you – you don't talk to anybody, they put you on hold (ID#12)* • *A young fellow came in and he seemed really dishevelled. The women were very busy behind the counter, but they treated him with so much respect and a good attitude. I thought it was amazing … I thought, yes, I'm in the right hands here (ID#19)* • *Initially, they gave me a timeframe of when it was gonna start and it kept getting put back and put back, so it took a lot longer to start than I thought it would (ID#05)*

### 3.1 Theme 1: Physiotherapy-led osteoarthritis services were acceptable to most participants

#### 3.1.1 Subtheme: An advanced practice assessment was acceptable to most participants, but often not strongly desired

Most participants were confident in the assessment, with a perceived thorough assessment contributing to its acceptability. A few participants preferred seeing an advanced practice physiotherapist instead of a surgeon, often associated with a desire to avoid surgery. Most, but not all, participants viewed it as an acceptable alternative to seeing a surgeon at the hospital.

*I think [the advanced practice physiotherapist]'s at a good level. I don't need to see a surgeon. I think[the advanced practice physiotherapist]knows what [they’re] doing (ID#13)*

However, not all participants were in favour of an advanced practice assessment. Several participants perceived the assessment as unremarkable, difficult to recall, and were unclear on its purpose.

A perceived superficial assessment (e.g. not being asked sufficient questions) was associated with a lack of acceptability for some participants. A few participants would have preferred to see a surgeon.

A couple of participants perceived the assessment as acceptable provided a pathway to orthopaedics was available. Most participants who desired seeing an orthopaedic surgeon had the intention of having surgery, yet a few appeared to lack trust in the advanced practice assessment and sought a surgeon's opinion only to provide reassurance of their future next steps. A few participants also desired imaging after finishing physiotherapy, perceiving imaging would help guide future management plans.

*I’d like to have some sort of x-ray or scan … I want to see exactly what’s happening in my knee instead of trying to just put Band-Aids on the problem all the time (ID#4)*

Of those participants who commented on being seen at a community centre instead of a hospital, all but one perceived the community setting favourably. Potential benefits of a community setting over hospital-based care including shorter waiting times and proximity to home.

#### 3.1.2 Subtheme: Exercise is perceived as beneficial and acceptable by most participants, including supervision from a physiotherapist and being in a group environment, but not often strongly desired

Most participants perceived exercise as acceptable, with improvements often attributed to an increase in strength. Some participants reported improvements in pain, while others reported improvements in function or confidence. Whilst most participants perceived some benefit, only a few were strongly in favour of exercise.

*The pain in my knee wasn't quite as bad as what it was (ID#10)*

In contrast, some participants were disappointed with an overall lack of improvement in pain or function, including a couple reporting flare ups which mostly settled.

The majority of participants valued the support of a supervising physiotherapist. Attending a group exercise class was perceived to have benefits for almost all participants, with perceived benefits including peer support and social interactions. Most participants reported difficulty with being motivated to exercise, and attending a group class was perceived by some participants as assisting with motivation.

#### 3.1.3 Subtheme: Participants views on education sessions were mixed

Views on education sessions were evenly split, with approximately half of participants reporting feeling they were beneficial. Being able to ask questions in the education sessions was valued.

However, approximately half of participants felt the education sessions were not beneficial, and several struggled to recall information. A few participants perceived their questions were unable to be answered, others felt the education sessions did not add to the program, with a couple not satisfied with the format of the delivery.

### 3.2 Theme 2: Physiotherapy care received was typically perceived as insufficient to fully address all ongoing knee osteoarthritis management needs

#### 3.2.1 Subtheme: Exercise is not perceived as a long-term solution

Some participants viewed osteoarthritis as a structural problem, with pain related to the structural changes. Exercise was perceived as not able to resolve or ‘cure’ osteoarthritis, even when symptoms had improved.

*I think it was a very good program and I felt good when I finished it. And I tried to keep up the exercises for a while, but underneath it, I still have the knee problem … Because you can do exercises forever, but if there’s a structural problem in there, it’s not gonna sort the problem out (ID#4)*

#### 3.2.2 Subtheme: Ongoing support (both exercise group and monitoring) was commonly desired by participants

Several participants stated that the GLAD® program should offer more sessions. Almost all participants reported continuing with exercises independently after finishing GLA:D® was challenging, and several desired ongoing support and monitoring. A number of participants expressed a desire for ongoing review and to have a definitive strategy for next steps in managing their knee issues.

#### 3.2.3 Subtheme: Uncertainty regarding future options and prognosis

Uncertainty regarding unpredictable joint symptoms, and future prognosis for their knee osteoarthritis, including timing of potential future surgery, was frustrating to some participants. Several participants reported being unsure of the ongoing management plan, including other management options, and a perception of being left on their own after completing physiotherapy or the GLA:D® program.

*I'm, kind of now, in a sense, abandoned… done the program now, so what next, no operation next, so what next, “Well, that's it. Thank you and goodbye.” (ID#16)*

#### 3.2.4 Subtheme: Surgery was often perceived as inevitable and the optimal treatment, although often a last resort

For most participants, surgery was perceived as the only option to fully resolve all their knee problems whereas others were accepting of some functional limitations. Some were keen for surgery imminently. However, most participants desired avoiding surgery for as long as possible and surgery was often seen as a last resort, although mostly due to potential risks of surgery, rather than viewing surgery as unnecessary. Although participants described family and friends experiencing mixed results after surgery, negative experiences predominated and contributed to some participants’ desire to avoid surgery. Comorbidities, previous negative experiences with other surgery, and age were reasons commonly cited for wanting to avoid surgery. Despite most participants wishing to avoid surgery, it was often perceived as the only option in the future if symptoms worsened. Many participants deemed surgery inevitable.

*I think, at some stage in my life, I will have to have a knee replacement, yes (ID#10)*

Repeating exercise programs, engaging in other forms of exercise, or other non-surgical strategies were rarely considered when participants were discussing options if symptoms worsened in the future. Only a few participants thought surgery could be avoided long term.

*No, [I don't think I will need to have a knee replacement in the future] definitely not, not at all (ID#15)*

### 3.3 Theme 3: Effective clinical and non-clinical communication was strongly linked to acceptability of physiotherapy-led osteoarthritis services

Participants who perceived physiotherapy-led osteoarthritis services as acceptable reported a strong therapeutic alliance and confidence in the clinician. Having access to a trusted physiotherapist and being able to ask questions improved participants’ perceived acceptability of the non-surgical care pathway.

*Because nine times out of 10, that's all it is. It’s just talking to someone who knows (ID#10)*

For others, a perceived disinterest or a cursory interaction from both physiotherapists and administrative staff was associated with lack of acceptability of care, and a lack of confidence in care for a few participants. Some participants were dissatisfied with communication from the health service, whereas others reported positive impressions of the health service. Inefficient organisational processes at health services were a source of frustration for a few participants.

In particular, delayed appointments contributed to dissatisfaction for a few participants.

*I felt I was nothing. I actually thought they'd forgotten about me (ID#12)*

## 4 Discussion

To our knowledge, this is the first study to comprehensively explore patient acceptability of community-based physiotherapist-led first-line care, including advanced practice physiotherapist assessment, for knee osteoarthritis. Shifting care away from hospital settings into community-based services was acceptable, suggesting a viable alternative option to overburdened hospital-based orthopedic assessment services. However, despite the advantages of a community-based model of care, we also identified several barriers to successful implementation. Many participants desired ongoing support and monitoring, struggled to recall information and self-manage following an exercise and education program; and were uncertain about how well physiotherapist-led care could address ongoing knee issues. An impairment discourse was prevalent, and surgery often viewed as inevitable. High quality communication and a strong therapeutic alliance were key to the acceptability of physiotherapy-led care. Our novel findings should be used to inform future implementation strategies and ultimately help alleviate pressure on hospital orthopaedic waiting lists.

Greater acceptability of physiotherapist-led services was strongly influenced by quality communication and person-centred care. Where communication was considered poor, acceptability was lower, reflecting previous research [33], and supporting communication and provision of person-centred care as core health professional capabilities [34]. Quality communication and person-centred care helps develop a strong therapeutic alliance [35]. Highlighting the importance of a strong therapeutic alliance, it can increase trust in physiotherapists [36], optimise pain and physical function outcomes [37], improve self-efficacy [38] and increase adherence to exercise [39]. Aligning with previous research [40], acceptability of care was also influenced by interactions with non-clinical staff and health service processes, suggesting empathetic communication should be prioritized across all staff roles.

Despite most participants attending two osteoarthritis education sessions, and all consulting an advanced practice physiotherapist, many struggled to recall information, desired more information, and were uncertain of future management options. This may reflect that around half of participants did not find the education provided valuable. Ensuring more comprehensive education and support focused on a participatory discourse [41] to address this apparent unmet need is important considering greater understanding and less uncertainty can facilitate uptake of treatment recommendations [42]. Quality education can also improve self-efficacy, and may reduce perceived need for surgery [43]. Further work is needed to understand if the limitations in education provided to participants in this study reflects the content and/or delivery of the GLA:D® program provided, or other factors. Offering information in multiple formats [44] and using methods such as the Teach-back method may improve knowledge retention and improve health outcomes [45].

A lack of motivation and confidence to exercise independently, and inadequate ongoing support, including a lack of access to ongoing exercise groups, were key barriers to long-term exercise participation. Addressing this is important, as improvements in pain and disability typically decline once people stop exercises [46]. Our participants mostly valued group exercise for motivation, and social and peer support. However, not all participants were confident to self-manage after attending supervised exercise sessions, despite guidelines recommending this [1]. Identifying who is likely to require or benefit from ongoing support is encouraged, and may consider factors known to influence self-efficacy and self-management engagement such age, body mass index, comorbidity, education level and health literacy levels [[47], [48], [49]]. Regardless, health service provision of accessible, ongoing support may assist patients to continue with non-surgical management in the long-term [50], reducing demand on hospital wait lists.

Aligning with previous qualitative research [5], surgery was commonly perceived as the required treatment to resolve symptoms and fix a structural problem by our participants, with exercise unable to resolve a biomechanical issue, indicating an impairment discourse [51]. This qualitative finding from our cohort also reflected that following physiotherapist-led care, most (n = 13/20) participants were willing for surgery or perceived surgery would be required. These impairment-focused beliefs, consistent with those previously reported by patients [5,42] and health professionals [6], reduced acceptability of physiotherapy-led care but are contrary to current evidence. Exercise does not appear to accelerate knee osteoarthritis disease progression [52], can improve pain and function [53], and most people manage their symptoms without requiring surgery [54]. Outcomes after total knee arthroplasty are varied [55], with up to 20% having ongoing pain after this surgery [56]. Work is needed to change the narrative of osteoarthritis management [57] and help improve the acceptability and uptake of exercise (and physiotherapy-led care) [58], and ensure people have realistic surgical expectations. Strategies including upskilling health professionals, public health campaigns and use of musculoskeletal champions [59] could assist in addressing misconceptions and reframing osteoarthritis as a condition responsive to non-surgical management. These may improve the acceptability of non-surgical care, and reduce unnecessary surgical demand and burden on public waiting lists.

### 4.1 Limitations and strengths

Several factors may have influenced findings from our study. We explored the experiences of people with knee osteoarthritis attending community-based physiotherapist-led care following referral for orthopaedic assessment in Australia. Our findings may not generalise to other settings or populations. However, a strength was purposively recruiting participants with different views on willingness for surgery, across multiple health services offering varying types of physiotherapist-led support, including some receiving privately funded and telehealth care, supporting the relevance of findings across diverse service provision. Our sample reflected the broader MOTION cohort and typical knee osteoarthritis population -with a higher proportion of females compared to males, older age, and increased BMI [60].

Participants had no out-of-pocket physiotherapy costs, consented to transfer of their hospital-based referral to a community health service and were reassured orthopaedic consultation would not be delayed if required; all of which may influence acceptability. Their views may not be representative of all people with knee osteoarthritis. Excluding participants not fluent in English may limit the generalisability of our findings. A separate qualitative analysis is being undertaken involving cultural and linguistically diverse participants, including those without English-language proficiency. All researchers were physiotherapists, but with diverse backgrounds, from a range of counties, academic roles and clinical settings. No pilot interview was conducted, however the research team refined the topic guide iteratively.

### 4.2 Conclusion

A community-based advanced practice physiotherapy clinic appears to be an acceptable alternative to hospital advanced practice clinics, supporting implementation to shift care out of hospital-based models. While physiotherapy-led care was mostly acceptable to patients, uncertainty remained regarding how well it addressed participants’ understanding of osteoarthritis and long-term knee issues, and an impairment discourse remained common. Prioritising support for long term management of people with knee osteoarthritis by physiotherapists is recommended. Clear communication and a strong therapeutic alliance increased acceptability and should be prioritized by clinicians and health services. This study provides important guidance on current challenges and enablers to the acceptability of community-based advanced practice and physiotherapy-led care, and may guide implementation of these services in other healthcare settings.

## Author contributions

*Study conception and design:* AJG (led), AME, JAW, JLK, NFT, CJB. *Collection and assembly of the data:* AME and JL. *Analysis and interpretation of the data:* All authors. *Drafting of the article* -AJG. *Critical revision of the article for important intellectual content* -all authors. *Final approval of the article:* all authors. *Obtaining of* funding-CJB.

## Role of the funding source

This work was supported by the Medical Research Future Fund from the National Health and Medical Research Council (NHMRC) grant number [APP1175374]. AME was supported by a Canadian Institutes for Health Research Post-Doctoral Fellowship. AJG is supported by an Australian Government Research Training Program (RTP) Scholarship paid to La Trobe University]. JL is supported by a La Trobe University Graduate Research Scholarship. The funders had no role in study design; in the collection, analysis and interpretation of data; in the writing of the report or in the decision to submit the article for publication.

## Conflicts of interest statement

AJG works for GLA:D® Australia program one day per week. AME is the national lead for GLA:D® Canada program. JLK and CJB Co-lead the not-for-profit GLA:D® Australia program. Time spent on this project is funded in-kind by employing institution La Trobe University. JLK receives honorarium as BJSM editor. JAW is a sessional tutor for the GLA:D® Australia program. All ICMJE Conflict of Interest Forms for authors are on file with the publication and can be viewed on request.
