# Supplementary material for: “A very good program … but I still have the knee problem”: A qualitative study exploring patient acceptability of physiotherapy-led osteoarthritis services
Source: Osteoarthr Cartil Open. 2026 May 5;8(2):100799. doi: 10.1016/j.ocarto.2026.100799 (PMC13199984; doi:10.1016/j.ocarto.2026.100799)
Supplement: Multimedia component 1 [file mmc1.docx]

COREQ Checklist

| Domain 1 Research team and reflexivity |  | Info | Where |
| --- | --- | --- | --- |
| Personal characteristics  1. Interviewer or facilitator | Which author/s conducted interview/focus group | Interviewers and background outlined in data collection | Lines 87-88, 94-98 |
| 2. Credentials | What were the researchers’ credentials e.g. PhD, MD | Credentials and background experience outlined in data and data analysis | Lines 94-98 103-108, 114-115 |
| 3. Occupation | What was their occupation at the time of the study | Physiotherapists and academics Listed in data collection and data analysis | Lines 94, 103-105, 107-108 |
| 4. Gender | Was the researcher male or female | Interviewers gender provided in data collection | Line 94 |
| 5. Experience and training | What experience or training did the researcher have | Interviewers-one experienced qual researcher provided training to PhD candidate (2^nd^ interviewer). Research team included experienced qualitative researchers. Qualitative research course. Listed in data collection and data analysis | Lines 94-94104-108, 114-115 |
| Relationship with participants 6. Relationship established | Was a relationship established prior to study commencement | No relationship with interviewers outlined in data analysis, one member of research team worked at recruitment site | Lines 97-98, 107110 |
| 7. Participant knowledge of the interviewer | What did the participants know about the researcher? E.g. personal goals, reasons for doing the research | Knew was part of larger MOTION study and aim of research as per PICF |  |
| 8. Interviewer characteristics | What characteristics were reported about the interviewer/facilitator e.g. bias, assumptions, reasons and interest in the research topic | Two interviewers, one PhD candidate, one experienced qualitative researcher. One member of research team (not an interviewer) worked in area being explored. Outlined in data collection and data analysis | Lines 94-94, 107-112, 115-117 |
| Domain 2 study design |  |  |  |
| Theoretical framework  9. Methodological orientation and theory | What methodological orientation was stated to underpin the study? | Interpretative description with interview guide based on Theoretical Framework of Acceptability | Lines 49-52, 88-90 |
| Participant selection  10.Sampling | How were participants selected? E.g. purposive, convenience, consecutive, snowball | Open to all eligible participants larger study with purposive sampling to ensure mix of views on willingness for surgery | Lines 55-58, 77-81 |
| 11. Method of approach | How were participants approached? E.g. face to face, email, telephone | Telephone as per participants section | Lines 72-75 |
| 12. Sample size | How many participants were in the study | Final Sample size 20 (reported in results) , continued until sufficient information power achieved then further two interviews to confirm | Lines 137-138 |
| 13. Non-participation | How many people refused to participate or dropped out? Reasons? | Three declined, three did not return consent form, and one not contactable after returning consent form -no reasons given | Lines 133-137 |
| Setting  14. Setting of data collection | Where was data collected? E.g. home, clinic, workplace | Remotely via zoom or telephone -outlined in data collection | Lines 87-88 |
| 15. Presence of non-participants | Was anyone else present besides the participants and researchers | No one else was present during interviews, all interviews were 1:1 |  |
| 16. Description of sample | What are the important characteristics of the sample e.g. demographics, date | Demographics collected outlined in data analysis and reported in tables 1 -summary and Appendix 2-individual | Lines 138-143, Table 1 and appendix 2 |
| Data collection  17. Interview guide | Were questions, prompts, guides provided by the authors? Was it pilot tested? | Interview guide developed described in data collection, included as supp file 1 | Lines 89-91 and Appendix 1 |
| 18. Repeat interviews | Were repeated interviews carried out? If yes how many | No |  |
| 19. Audio/visual recording | Did the research use audio or visual recording to collect the data | Audio recorded-outlined in data collection | Lines 91-92 |
| 20. Field notes | Were field notes made during and /or after the interview or focus groups | Yes, described in data analysis | Line 114- |
| 21. Duration | What was the duration of the interviews or focus group | 14-56 minutes Outlined in results | Line 144 |
| 22. Data saturation | Was data saturation discussed? | Yes-sufficient information power discussed in data collection | Lines 98-100 |
| 23. Transcripts returned | Were transcripts returned to participants for comment/and or correction? | No |  |
| Domain 3 analysis and findings |  |  |  |
| Data analysis  24. Number of data coders | How many data coders coded the data | Two for each interview, a third read transcripts | Lines 119-127 |
| 25. Description of the coding tree | Did authors provide a description of the coding tree | No Coding tree but themes and subthemes described in results |  |
| 26. Derivation of themes | Were themes identified in advance or derived from the data | Themes were derived from the data | Lines 119-130 |
| 27. Software | What software, if applicable, was used to manage the data | NVivo was used by one of the coders | Lines 122 |
| 28. Participant checking | Did participants provide feedback on the findings | No |  |
| 29 Quotations presented | Were participants quotations presented to illustrate the themes/findings? Was each quotation identified e.g. participant number? | Yes -examples included throughout results, 3 and appendix 4 contain further examples | Lines 165-166, 178-179, 191, 215-218, 233-234, 248, 253-254, 262, 270, Table 3, appendix 4 |
| 30. Data and findings consistent | Was there consistency between the presented data and the findings | Yes-see results with exemplar quotes, and table 3 and appendix 4 for further example quotes | Lines -148-270, table 3, appendix 4 |
| 31. Clarity of major themes | Were major themes clearly presented in the findings | Yes; see results for overarching themes and subthemes | Lines 148-152, 158-160 185-186, 201, 209-11, 220-221, 227, 236-237, 256-257 |
| 32. Clarity of minor themes | Is there description of diverse cases or discussion of minor themes | Yes, throughout results section Examples of diverse cases included and outlined in results, Table 3 and Appendix 4 | Lines 270, table 3, appendix 4 |
