# Supplementary material for: “A very good program … but I still have the knee problem”: A qualitative study exploring patient acceptability of physiotherapy-led osteoarthritis services
Source: Osteoarthr Cartil Open. 2026 May 5;8(2):100799. doi: 10.1016/j.ocarto.2026.100799 (PMC13199984; doi:10.1016/j.ocarto.2026.100799)
Supplement: Multimedia component 2 [file mmc2.docx]

**Appendix 1**

**Interview topic guide**

Acceptability of prior/current Knee OA management

Now I want you to think back to before you had the initial appointment at the clinic.

1. Can you start by telling me a bit about your knee and the treatments you had beforehand?
*-what prompted you to first seek help for your knee?
-What treatments have you had since your knee started bothering you? (e.g. what different people such as doctor, physio, surgeon) have you seen for your knee?)*

2. Please describe what it was like getting an appointment with the people you wanted to see about your knee before taking part in the study?

3. To what extent were you satisfied with the treatment(s) you had received for your knee?

4. Who do you feel is the most important person (profession) to advise you about your knee and why?

Acceptability of access to community health service OAHKS Initial Appointment:

5. Please describe the process of getting an appointment at the community health service (may need to name the service) for your knee?

6. What were your expectations of your appointment prior to attending the community health service?

7. What was it like trying to make an appointment? *(eg. waiting times, knowing who you were going to be seeing, My Aged Care if aged 65 or over, additional information you needed to provide to the centre, anyone else you needed to speak to, understanding of referral pathways)*

8. What has made it hard for you to get to the centre and to see someone?

9. What has/could make it easier for you to get to the centre and to see someone?

Acceptability of Attending OAHKS

Now I want you to think about the appointment you received at the community health service.

10. What are your thoughts about being seen at a community health service instead of in a hospital? *Would you prefer to have had your first appointment/assessment at a hospital or a community health centre? Can you tell me why? What advantages do you feel there are to seeing someone at the hospital/CHS (depending on their response)? What disadvantages do you feel there are to seeing someone at the hospital/CHS (depending on previous response)*

11. Can you share your experience of the initial assessment with the physiotherapist?
-To what extent do you feel your expectations were met? *(use as prompt if needed…. thoughts on not seeing a surgeon)*  -How useful did you find this appointment?
-How satisfied were you with this appointment?

Acceptability of care/management plan

12. Can you describe/ recall the recommendations that were made at the first assessment/appointment?

13. How happy were you to follow the recommendations/plan from the first assessment?

14. What did you think about this management plan? (*may include referral to ortho, advice to see GP, seeing care coordinator)*

15. How many of your appointments were you able to attend?

-Can you share with me which appointments these were? (eg OAHKS vs GLA:D vs dietitian)

-were there any services you decided you didn’t want to see (eg. dietetics); *or I understand that you declined seeing a dietitian…can you tell me abit more about that?*

16. If you were referred to GLA:D, what did you think about being referred to the GLA:D program?

*How confident were you that you could participate in the GLA:D program?*

Now thinking about your participation in the GLA:D program, you attended (if they did)

18. How did you feel about the GLA:D program after you had finished going to GLA:D?

*Prompt- -how did you feel after participating in GLA:D?*

Depending on response: (may have already been answered)
 what helped you to attend?
 what stopped you attending/made it hard to attend? (*eg. time, language, wasn’t feeling benefit, transport difficulties, don’t like exercise, motivation, beliefs around exercise, work, family commitments*)

19. What could make it easier for people to attend? *(eg parking, out of hours, transport, times offered, confidence/belief in exercise)*

Acceptability of participation in MOTION study (for separate process evaluation)

Now thinking about taking part in the research study:

20. Can you tell me about your experience in taking part in the research study?
-Why did you decide to participate in the study? *(eg. free GLA:D, didn’t want surgery, shorter waiting times to see someone).*

21. What were your expectations from participating in the study?
-What, if any, were your concerns about taking part in the study?

22. How satisfied/happy are you with the care you received? (*May have already been answered with earlier questions-but prompts could include referral to orthopaedics, other health professionals eg podiatry, seeing the care coordinator)*

21. What, if anything, made it difficult for you to participate in the study *(may halve already answered lot of this-also consider answering questionnaires, understanding the process)*

22. What, if anything, made it easier for you to participate in the study? *(eg free treatment)*

23. Overall, can you tell me how useful you have found taking part in the study for your knee problem?

24. What do you feel needs to happen now with your knee? *Eg. How happy are you that you can look after your knee yourself? What are your thoughts on whether you are likely to need or want a knee replacement within the next 6 months? 12 months? 2 years?*

Is there anything else you would like to add or talk about?
