# Supplementary material for: “A very good program … but I still have the knee problem”: A qualitative study exploring patient acceptability of physiotherapy-led osteoarthritis services
Source: Osteoarthr Cartil Open. 2026 May 5;8(2):100799. doi: 10.1016/j.ocarto.2026.100799 (PMC13199984; doi:10.1016/j.ocarto.2026.100799)
Supplement: Multimedia component 3 [file mmc3.docx]

| **Appendix 2**  Table 1: Individual participant characteristics | | | | |  |  |  |  |  |  |  |
| --- | --- | --- | --- | --- | --- | --- | --- | --- | --- | --- | --- |
|  | | | | |  |  |  |  |  |  |  |
| **ID** | **Age (years)** | **Sex** | **BMI (kg/m^2^)** | **Work status** | **Country of birth** | **First language** | **Indigenous status** | **Willing for surgery^a^ baseline** | **Future need for surgery^b^ baseline** | **Willing for surgery**  **(4 months^c^)** | **Future need for surgery**  **(4 months)** |
| 1 | 64 | F | 36.1 | Not working; knee related^d^ | Australia | English | Neither Aboriginal nor Torres Strait Islander | Yes | N/A | Yes | N/A |
| 2 | 73 | F | 21.1 | Retired | Australia | English | Neither Aboriginal nor Torres Strait Islander | No | No | No | Yes |
| 3 | 73 | F | 36.7 | Retired | Australia | English | Neither Aboriginal nor Torres Strait Islander | Yes | N/A | Unsure | No |
| 4 | 77 | F | 26.5 | Retired | Australia | English | Neither Aboriginal nor Torres Strait Islander | Yes | N/A | Yes | N/A |
| 5 | 72 | M | 26.2 | Retired | Australia | English | Neither Aboriginal nor Torres Strait Islander | Yes | N/A | No | No |
| 6 | 71 | F | 25.3 | Retired | Australia | English | Neither Aboriginal nor Torres Strait Islander | No | Yes | Unsure | Yes |
| 7 | 78 | M | 26.5 | Working P/T^e^ | Australia | English | Neither Aboriginal nor Torres Strait Islander | No | Yes | No | Yes |
| 8 | 68 | M | 30.5 | Working^f^ | Australia | English | Neither Aboriginal nor Torres Strait Islander | Yes | N/A | No | Yes |
| 9 | 70 | F | 27.8 | Retired | Australia | English | Neither Aboriginal nor Torres Strait Islander | Yes | N/A | Unsure | No |
| 10 | 69 | F | 30.7 | Retired | Australia | English | Neither Aboriginal nor Torres Strait Islander | No | Yes | Unsure | Yes |
| 11 | 58 | F | 36.7 | Working | Australia | English | Neither Aboriginal nor Torres Strait Islander | Yes | N/A | No | Yes |
| 12 | 81 | F | 28.6 | Retired | Italy | Italian | Neither Aboriginal nor Torres Strait Islander | No | No | No | Yes |
| 13 | 47 | F | 27.3 | Working P/T | India | Malayalam | Neither Aboriginal nor Torres Strait Islander | Unsure | Yes | No | No |
| 14 | 63 | M | 31.8 | Working P/T | Australia | English | Neither Aboriginal nor Torres Strait Islander | No | Yes | No | No |
| 15 | 59 | F | 31.9 | Working | Indonesia | English | Neither Aboriginal nor Torres Strait Islander | No | No | No | No |
| 16 | 85 | M | 27.0 | Retired | United Kingdom | English | Neither Aboriginal nor Torres Strait Islander | Unsure | Yes | Unsure | Yes |
| 17 | 89 | M | 22.5 | Retired | Australia | English | Neither Aboriginal nor Torres Strait Islander | No | No | No | No |
| 18 | 60 | M | 30.7 | Not working; knee-related | Australia | English | Neither Aboriginal nor Torres Strait Islander | Yes | N/A | Yes | N/A |
| 19 | 75 | M | 24.8 | Retired | Austria | German | Neither Aboriginal nor Torres Strait Islander | Yes | N/A | Yes | N/A |
| 20 | 71 | F | 37.9 | Not working^g^ | Australia | English | Neither Aboriginal nor Torres Strait Islander | No | No | No | Yes |

BMI= Body mass index; a = willing to under total knee replacement within next week; b = Response to question “Do you think you will need knee replacement surgery at any time in the future?“; c = 4 months after baseline questionnaires; d= Not working due to knee osteoarthritis; e= working part time (<30 hours a week); f = working full time (≥30 hours a week); g= not working due to reasons other than knee;

Table 2: Individual osteoarthritis management received

| **Participant ID** | **Individual physio**  **sessions^a^** | **GLAD exercise  sessions^b^** | **GLA:D education sessions^c^** | **Location of GLA:D^d^** | **Tele-health GLA:D^e^** |
| --- | --- | --- | --- | --- | --- |
| 1 | 5 | 0 | 0 | CHS | No |
| 2 | 3 | 11 | 0 | CHS | No |
| 3 | 0 | 10 | 2 | CHS | No |
| 4 | 0 | 11 | 1 | CHS | No |
| 5 | 0 | 12 | 1 | CHS | No |
| 6 | 0 | 12 | 2 | CHS | No |
| 7 | 0 | 12 | 1 | CHS | No |
| 8 | 0 | 12 | 2 | CHS | No |
| 9 | 4 | 12 | 2 | CHS | No |
| 10 | 0 | 12 | 2 | CHS | No |
| 11 | 3 | 5 | 2 | CHS | No |
| 12 | 6 | 12 | 2 | CHS | No |
| 13 | 0 | 11 | 1 | CHS | No |
| 14 | 1 | 6 | 0 | PP | Yes |
| 15 | 0 | 12 | 2 | CHS | No |
| 16 | 1 | 11 | 1 | PP | No |
| 17 | 0 | 11 | 2 | CHS | No |
| 18 | 0 | 12 | 2 | CHS | No |
| 19 | 2 | 12 | 2 | PP | No |
| 20 | 2 | 12 | 2 | CHS | No |

CHS = Community Health Service; GLA:D = Good Life osteoArthritis: Denmark; N/A = Not applicable; PP = Private Practice; a = Number of one to one physiotherapy sessions attended (GLA:D exercise sessions); b = Number of GLA:D exercise sessions attended; c = Number of GLA:D education sessions attended; d = service location where attended GLA:D ; e = Attended GLA:D via telehealth
