# Supplementary material for: “A very good program … but I still have the knee problem”: A qualitative study exploring patient acceptability of physiotherapy-led osteoarthritis services
Source: Osteoarthr Cartil Open. 2026 May 5;8(2):100799. doi: 10.1016/j.ocarto.2026.100799 (PMC13199984; doi:10.1016/j.ocarto.2026.100799)
Supplement: Multimedia component 4 [file mmc4.docx]

Appendix 3

**Table 1 Four-month willingness for surgery**

|  | **Interviewees n=20** | **MOTION Cohort n=300** |
| --- | --- | --- |
| Willingness for joint replacement now^a^ percentage (number)  Yes  Unsure  No | 20 (4)  25 (5)  55 (11) | 58 (173)  15 (45)  27 (82) |
| Need for future joint replacement surgery^b^, (percentage)^c^ number  Yes  No  Total perceiving surgery required percentage (number) | 56 (9)  44 (7)  65 (13) | 62 (78)  38 (48)  78 (235) |

a = Willingness to undergo Total Knee Arthroplasty within the next week; b = Response to question “Do you think you will need knee replacement surgery at any time in the future?” c=percentage of those who responded no or unsure to surgical willingness now question
