# Supplementary material for: “A very good program … but I still have the knee problem”: A qualitative study exploring patient acceptability of physiotherapy-led osteoarthritis services
Source: Osteoarthr Cartil Open. 2026 May 5;8(2):100799. doi: 10.1016/j.ocarto.2026.100799 (PMC13199984; doi:10.1016/j.ocarto.2026.100799)
Supplement: Multimedia component 5 [file mmc5.docx]

Appendix 4

Table 1: Additional quotes supporting Theme One: Physiotherapy-led osteoarthritis services were acceptable to most participants

| **Subtheme** | **Supportive quotes** |
| --- | --- |
|  |  |
| An advanced practice assessment was acceptable to most participants, but often not strongly desired | *I could tell [the advanced practice physiotherapist] was on top of everything. I could see that. (ID#3)*  *I think it was very good because we did things like squatting or [the APP] was moving my leg, the flexibility of it, so I thought it was pretty thorough to know what status I was in. (ID#18)*  *[The advanced practice physiotherapist] had a lot of questions and I answered all those questions, and they did a thorough examination of the knee and put me through some tests . . . [The advanced practice physiotherapist]’s obviously a very experienced physio and very switched on (ID#8)*  *No, I felt fine [about seeing physiotherapist instead of surgeon]. (ID#14)*  *[The advanced practice physiotherapist] was helpful in terms of explaining different things about the condition, also explaining what options were available in terms of any surgical interventions if needed or that, you know, if I didn't want to go ahead with that straightaway that I could still make contact and book in at a later time if I felt that I may need to want to proceed.(ID#11)*  *I was happy with that [seeing advanced practice physiotherapist]. I prefer that. If there was a program designed to give you more longer use of your legs without having an operation, I’d prefer to do that than go straight to surgery. (ID#5)*  *Because it’s all a pathway to the orthopaedics, isn’t it? I’m assuming it is. As a layperson, I’m assuming it all goes back to them. (ID#1)*  *It was part of the study, and I was going there as how I interpreted – I was going there as part of what I'd signed up for with the GLA:D program. It wasn't actually to assess me or find out anything much from – it was GLA:D focused rather than me focused (ID#16)*  *He didn't really touch me . . . It was positive, but we didn't do much. We didn't talk much . . . I didn't get much out of it . . . [the advanced practice physiotherapist] wasn't . . . reassuring (ID#12)*  *Well, I think it [the advanced practice assessment] was more useful for the GLA:D program than for me, because it could have been directed straight away. . . So it was more beneficial for the program than for me. (ID#19)*  *A surgeon on the operating table, please, just get it over with. (ID#1)*  *I just came away feeling it was a typical first appointment and I wasn’t disappointed. I wasn’t excited or anything about it. (ID#3)*  *Well, I would be very interested now to see whether the gains I have made, how that would translate into what next. I hadn't ruled out surgery at all in my own mind but I was really very interested and keen to see what a surgeon would actually say. So yes [have seen a surgeon]. Definitely.. (ID#16)*  *And I guess, from my perspective, the only other thing that I would like to see, because I think August is my one year that I’ve been there, is that whether a new x-ray or whether there's some way that [the advanced practice physiotherapist] could – if you could do that, so [the advanced practice physiotherapist] could say, well, it's got worse, it's got better, it hasn't changed, I think I'd like to know that. I'd like to know, like, do I have to go to my doctor to do that or could [the advanced practice physiotherapist] say to me, “Let's do another x-ray and see how it's going” or whatever. Yes, if there are changes. Well, if there are changes to it, I need to understand are they better or are they worse? (ID#10)*  *I think it [waiting time for advanced practice assessment] was better than what I expected. It was pretty quick. Usually, to get a specialist appointment you have to wait months (ID#13)*  *But when the program was offered to me sort of locally, it’s obviously much easier to get somewhere locally (ID#6)*  *I would have expected to be a bit wary of the hospital and expect the community health centre to be better in whatever way I see better. But it probably wasn’t like that, which surprised me (ID#6)* |
| Exercise is perceived as beneficial by most participants, including support of a group environment, but often not strongly desired  Participants views on education sessions were mixed | *The exercise has helped it. It’s built up the muscles around the knee . . . I can feel the strength in the knee building up and it makes – it’s logical that to build up the strength, I’m putting less pressure on the knee. (ID#8)*  *I felt it really helped me strengthen it. I don't – now I feel quite good with the knee. I don't have really any pain, except occasionally I might get a little twinge going up steps or something like that. But like I said, I'm pushed back riding, walking. I'm not really in any pain other than an occasional twinge, which I was quite sore and painful for quite a few months there. (ID#14)*  *Because I felt when I started the exercises, it improved my physique and the pain level, that there was no pain after that (ID#19)*  *I actually suffer one year . . . After I joined GLA:D, I got well. It’s so good, this program. . . this program is really helpful and I wish that you all have more funding to do it to impact more people and to offer this to more people. That will be a blessing to other people. (ID#15)*  *Well, it wasn’t too bad actually ‘cause it was only twice a week and they encouraged people to do the exercises at home, which I did. I felt more motivated, that’s for sure. And I was walking more and it wasn’t hurting me as much (ID#9*  *It gave me that ability to complete things like that without the sort of pain in my knee. I still have the pain but not the same sort of pain. (ID#10)*  *There was no sort of real discussion of will this get rid of my pain or anything like that. It was more just to say that I guess to enhance the muscle around the knee, in the hope that maybe the pain would go(ID#10)*  *I guess it didn’t have the more positive outcome that I would have hoped. (ID#6)*  *But it’s disappointing when you go through it and then find that it’s just the problem is still there. (ID#4)*  *I somehow feel like I don't think I made much improvement . . . I think it was, like I said, including one session that I had some issues with my knee, I think that's what it – maybe until then it was going good. My flexibility was a bit better than after that knee, whatever happened, and then it didn't improve. It just went on like, more like I'm trying to just finish the classes (ID#13)*  *Probably just the encouragement from [the physiotherapist]. We had a few, a couple of sessions where there was a few of us that were doing exercises, just sitting and talking and she'd ask questions. I think that was beneficial to hear how others are going and that, I think, in a way helped motivate. (ID#14)*  *Yeah, it was good. I thought it was good, because in a way, you encourage one another, you know? Put it this way, I probably was the older person there . . . Encouraging one another. And you think, “Oh, gee, I'm alright, there’s a lot worse.” (ID#12)*  *Look, I always think that if you're going to a particular class somewhere and then you've got a group of people that you're interacting with, that there's more incentive to go. (ID#11*  *The challenge is how on earth do I actually find the motivation and the oomph to do enough physiotherapy to stay fit enough. I'd much rather sit and have a cup of coffee or smoke a cigar or something, . . Because I found a physiotherapist at [the health service] so motivating, I thought I would go back to her on a private basis and I'm going actually once a fortnight. Obviously it isn't enough to make up for not doing anything for the other 13 days, which is more or less what happens (ID#16)*  *Some of the things I thought was really good was that it was quite small, so you’re getting the attention that you needed. . . the exercises could be described to you and demonstrated pretty much to you and there was enough time for the instructors to be available for you. (ID#4)*  *It was very good. The group was, they were lovely. I really enjoyed that. (ID#20)*  *Look, I always think that if you're going to a particular class somewhere and then you've got a group of people that you're interacting with, that there's more incentive to go. (ID#11)*  *I’ve enjoyed the whole thing. (ID#8)*  *I mean, I’m a very sociable person, but no, it [being part of a group] wouldn’t be the reason I went, or maybe put me off or attract me particularly. (ID#6)*  *Well, it [the social aspect] probably was a bit limited. You didn't really get introduced to other people, they’re just sort of, like, someone might come and say, "Oh, look, I’m Bill, you know, and how long have you been doing this" or something like that. I think it was good in the sense that sometimes, you know, in the early stages, I forgot exactly how to do the exercises, someone else was doing it, so you just copy them. So, in that sense, you didn't feel embarrassed or awkward. . . So, for the social aspect, it wasn't so much talking with other people, although you know one or two folks, we kept meeting them, so we kept talking about old cars or something like that as well as talking about the exercises. So I don't think that was – I mean, possibly, that sort of thing could be improved, but we've only got a limited time, really, we've only got an hour or something (ID#07)*  *When the first session came around, I was still in two minds about the operation. But then with the education and the exercises helping, I thought, “No, I don’t really need it.” . . . I think the information that they passed on was quite good. (ID#9)*  *They're very informative, that was good. (ID#20)*  *There was information related to exercise. I don’t know, it just resonated with me more.. . . I didn't expect it to resonate so much. I attended because I had to and because it was important to, and so I didn't expect to get as much value out of it as I did. So that was a pleasant surprise. (ID#11)*  *And I think what it really did was it redirected me to how much capacity you have as an individual to self-manage a lot to do with the condition and that you can influence that quite significantly. (ID#11)*  *I was able to ask a couple of questions, like did the cartilage really wear away or – and they said, “No, there’s always a little bit left.” So, I was able to clarify that. I tended to believe the physio because they seemed to know what was going on with the knee or even though they hadn’t seen the x-rays, but because it started overseas and it was brought to Australia, and they’ve had positive results from overseas. So, I thought, “Well, they must be doing something right.” So, I was quite happy with it (ID#9)*  *I think I did them [education sessions] at the start. I did one week the first week and I think the second one the next week, which I thought was good because that gave me more confidence and educated me on, “Well, I think I'll beat up the exercise there,” no, it was very good. (ID#18)*  *I found them [education sessions] actually a waste of time in a way, because I was told the same thing when we had those little interviews when I started off (ID#19)*  *Not for me totally convincing in the sense that the physiotherapist was charged with taking one through a range of, “Do you understand this? What about that? And here's something else,” sort of thing. I felt it was something that the physiotherapist was required to do as part of the GLA:D program rather than that it was something that I found really valuable. (ID#16)*  *I know what we need to do and all I know, but it is more like what I have to do for my knee was my main concern. You know, what can I do to improve my knee? Like, although there are things like moving and lubricating and all those things, it's just, we know that. But what can I do for my knee was my concern. (ID#13)*  *Yeah, it was basically the same thing as you’d get in the class (ID#5)*  *One thing I since learned listening to the health report is . . . I’m in my early 70s and if I had had an operation, it probably would only last for ten years, say. And if you can delay having your operation if you need one, as long as you can, that gives you – means you don’t have to have another operation basically . . . So, that wasn’t explained to me in the GLA:D program I think they could make that a little bit more clear as to why it’s beneficial to do this now ‘cause it delays – and the reason for delaying. (ID#5)* |
|  |  |
| GLA:D = Good Life osteoArthritis Denmark | |

Table 2 Additional quotes supporting Theme Two: Physiotherapy care received was typically perceived as insufficient to fully address all ongoing management needs for knee osteoarthritis

| **Subtheme** | **Supportive quotes** |
| --- | --- |
|  |  |
| Exercise is not perceived as a long-term solution | *There was no sort of real discussion of will this get rid of my pain or anything like that. It was more just to say that I guess to enhance the muscle around the knee, in the hope that maybe the pain would go,(ID#10)*  *The x-ray people plus [the advanced practice physiotherapist] also mentioned the fact that my kneecaps didn't align and that my pain is, because my bone is very close to rubbing on another bone. (ID#10)*  *I’d like to have some sort of x-ray or scan or something just to see what’s going because I haven’t had one of those for years(ID#4)*  *I somehow feel like I don't think I made much improvement prior to that and after that. I think it was, like I said, including one session that I had some issues with my knee, I think that's what it – maybe until then it was going good. My flexibility was a bit better than after that knee, whatever happened, and then it didn't improve. It just went on like, more like I'm trying to just finish the classes. . . I don't know what it is, but I wouldn't want to, unless I really need to do physio, I wouldn't go back to physio. (ID#13)*  *I followed the GLA:D program, the way I was supposed to, and I didn't let pain bother me. I just tried to get over that, to get used to it. . . it just got worse and worse. So I thought I go back to my doctor who I had been with almost 40 years and he said, “Look, you better go and see somebody,”*  *I guess I was just a bit disappointed that it didn’t have the same positive effect. It certainly wasn’t – you’re not going to damage yourself doing a series of exercises. They can only be good for you. But we all want a miracle cure and a miracle outcome. We can’t cure arthritis, but – I don’t consider it a waste of time and it wasn’t a bad experience, but I guess it didn’t have the more positive outcome that I would have hoped. (ID#6)*  *Pretty good because it was clear to me before the end that it actually was having a real impact. I think the first thing that I certainly understood was that I wasn't limping as much and then after time, that I wasn't limping at all. That was, for me, a really good measure, something must be happening. (ID#16)*  *My knee is never gonna be perfect, but if it stops you from ever having the surgery, I’m rapt. (ID#8)*  *Strengthen the knees, strengthen the leg, the muscle around it, so that you can manage the pain and then you don't have to have surgery. They emphasise that a lot. (ID#15)*  *I think most people would think if there's got arthritis or it's painful, I better not overwork it or do too much because I'm going to make it worse or I'm going to be in pain, but I realised that doing moderate or exercise what you can manage is actually good for you (ID#18)* |
| Ongoing support (both exercise group and monitoring) was commonly desired by participants | *I mean, it was twice a week for six weeks and I think I probably could have even gone on for longer than that. (ID#4)*  *As I said, I think the program is good and I think there is a lot of good in the program and, yeah, as I said, the only thing I would say is that once you've completed the program and you do continue, just to keep monitoring it (ID#10)*  *What I actually need is some bit of system or somebody to say, “Hey, hang on, don't give up, keep going, and it's worth trying this.” I mean I'm asking for a bit of system, which doesn't exist, I suppose. I would have thought there must be other people with the same notion that has been marched to the top of the hill (laughs)- (ID#16)*  *I was really happy with the [hospital advanced practice physiotherapist] they reviewed me every nine months or year and I felt like – well, if I did suddenly deteriorate rapidly, they were able to get me up the list. So, I guess that was what I would have preferred, something like that (ID#6)*  *I'm pretty confident [doing exercise at home] because I've been doing it here. (ID#18)*  *Yes, I'm pretty confident now. Like I said, my knee is doing really well. I'm able to walk up and down steps quite okay now. And I'm not really feeling any arthritis pain, which is great. I've still got a bit of soreness in the quad, where the tear was. But the arthritis, I'm not feeling any of that. So I'm really feeling quite positive about it. And I feel I can continue to manage it*. *(ID#14)* |
| Uncertainty regarding future options and prognosis | *[Asked about] plans to go overseas in a year’s time and I said, “Hmm, not sure.”*  *I don’t know. I mean, I’m doing everything that I’m doing. I don’t know what’s next (ID#1)*  *mean if one were part of us reporting procedure, or a check-up now and then, every three months or something. I don't know. I mean obviously if you're in a structure to give shape and purpose, or help give shape and purpose, but I’m-. As it is, just hugely grateful for all that I've been given, so I'm not actually asking anybody for more. I'm just explaining that I'm, kind of now, in a sense, abandoned.(ID#16)*  *Over time, I would expect that the condition of the knee would deteriorate and that, potentially, I might have greater levels of pain and need more pain medication. And that would be the point at which I'd potentially be considering what options are. But there isn't – like, it sort of seems that there doesn't seem to be much, like, in terms of any stem cell replacements or, you know, those kinds of things don't seem to be available or discussed, or I don't even know if that sort of thing works with this kind of condition. . . Like a cortisone injection or something like that. “Where is it?” Like, there's no discussion about that. The GLA:D program talks about exercise. But, you know, if that worsens, what are the potential nonsurgical options that are available? Like, I don't know what they are. (ID#11)*  *You do all the exercises and meanwhile, you're not on the list to have your knee done . . . by the time you get to 90 and your knee’s giving you trouble, it's the point where you don't want to have an operation either because it is a major operation. So, in a sense you know it's catch 22. (ID#7)*  *I wouldn't mind seeing a surgeon at the hospital and see, well, is it going to stay that way? If it stays the way it is, I can live with it. You understand? If it gets worse, as I get older, and what is it all, the procedure? (ID#12)*  *Having the psychological understanding of what’s going on with your knees and having a clear [idea of] what you can expect and what you can’t, and I think that helps too (ID#5)*  *Some of the things implied that I might not have to have an operation, but if I have to have an operation, then you will provide me with address or with a name, or whatever, for a surgeon and things like that, so I didn't feel alone. (ID#19)* |
| Surgery was often perceived as inevitable and the optimal treatment, although often a last resort | *GP just manages the pain and we keep going up and up and up and up and up with the medications, then we withdraw them, then we try something else. We go up and up and up with them, and we get off them and go on to something else, because nothing seems to be giving long-term pain relief. It’s all interim . . . But I’m just frustrated because it’s been so many years now, and now that the decision has been made by the medical profession, now it’s just the waiting now – the physical waiting of getting on the operating table. (ID#1)*  *So I had my knee done . . . I'm surprised how well it went and I just was hoping that I don't have to have it, but I did. And I'm glad I did because I couldn't have kept on going. (ID#19)*  *I actually find, I don't mind putting up with a bit of pain, I suppose, is the bottom line. I don't expect to be pain-free at my age. (ID#16)*  *I would love to have a complete knee recovery where I could do anything, but I think that’s probably unrealistic. So, I’m just happy that on the normal everyday things, that I’m not hindered. (ID#3)*  *I can walk all day. I haven’t got – part of my aims was – I used to surf a lot and part of my aim was to surf again. But I just haven’t got the spring in my legs that I used to have when I was younger, so I don’t think I will. . . . I’ve got enough strength in my legs to do everything I wanna do. (ID#5)*  *If I can use my knees and my legs to do what I wanna do now, I’m happy to put off surgery until I get to the point where it’s become such a difficulty that I can’t. And also, you’re kind of out of action for quite a while having the surgery ‘cause you got to have both knees done, so they do one and then they do the other one afterwards from what I understand. And so, that’s a few months out of your life, which if I can avoid it, I will . . . If I needed to, yeah. If I couldn’t walk and if it was too painful and stuff, I would repursue that [knee replacement] (ID#5)*  *But I wanted to do something to avoid having a knee replacement at my age. I know I'm 68 but I really didn't want to, you know, if I could live a bit more as it was, that was my aim. . . Most other people of my mum's friends who had knee replacements, most of them were reasonably happy so it wasn't that I felt that it was not going to be successful. It was just more, I guess I didn't – I'm not a fan of hospitals or operations, so a little bit of that. Yeah, and just within myself I wanted to give myself an absolute good chance to say “Okay. Well, no, I can't do you anymore. I really do need to replace the knee.” (ID#10)*  *I've had two ankle surgeries, one of which the initial one did not go well and so I've been left with partial nerve damage in my foot and had to have revision surgery. . . So, yeah, you always take a risk with it. So, if you don't need anything invasive, then my preference is not to do it. (ID#11)*  *Well, somehow the nerve on the outside of my knee was damaged [contralateral total knee replacement] and I’ve got no feeling on the outside of my knee. And I can’t kneel on it. And I thought, “No, I don’t wanna go through that again.” So, that’s why when they offered me the [Community Health Service] program, I just said yes to it. (ID#9)*  *I’d only say yes to surgery if I was totally convinced that it was my real choice. It was the only choice I had (ID#3)*  *So that's why if I can avoid the operation, I will because I'm old (ID#12)*  *I still see that as a probability, but I’d like to hope that we’ve delayed this by many, many years. So, if I’m in my mid-70s now, I hope it’ll be at least another ten years that I can hope to avoid a knee replacement. (ID#2)*  *Definitely I don't think about it [having a knee replacement], totally. Because now my knee is very good already. I can dance. I can do my gardening. (ID#15)*  *No, I'm sure it [knee replacement in the future] won't happen. (ID#17)* |
|  |  |

GLA:D = Good Life osteoArthritis: Denmark

Table 3 Additional quotes supporting Theme 3: Effective clinical and non-clinical communication was strongly linked to acceptability of physiotherapy-led services

| **Supportive quotes** |
| --- |
|  |
| *Well, with XX community health service, I’ve been very, very satisfied. I think they’re wonderful. So, they helped me. They give me concession price. Every person there is just very good. I’m very happy with them. (ID#3)*  *The supervising staff of the GLA:D program itself were acutely in touch with my specific needs and were so considerate on that basis of really trying to make it as straightforward as possible and highly successful for me to participate in. (ID#2)*  *Very welcoming. They made me feel welcome and it was really good, just comfortable, and just gave me the confidence to just start off with the exercise that they gave me. (ID#18)*  *The advanced practice physiotherapist] is really dedicated to their role there, I can see that. [The advanced practice physiotherapist] has the heart to help others. (ID#15)*  *I was quite shocked because the physios that I saw at the community health centre, I really was very unimpressed. (ID#6)*  *Just the knowledge and the ability to relate to me and – yeah. It’s a little bit odd. I mean, I just – they just didn’t seem to be on purpose (ID#6)*  *Look, it was very good. I was at [XX CHS} and they’re always friendly and helpful and was good access. (ID#5)*  *I'm only used to services Australia's way of doing things which was rather terrible, and I didn't expect much, but just the very opposite was true. Every person I dealt with knew what they were talking about. They're extremely pleasant to people. I mean I've heard of talking on the phone to somebody who obviously seemed to have a disability of some sort but are very gentle and incisive, and then also the training, everything was so good. (ID#19)*  *I was surprised that I . . . was looked after in such a good way, and it was great. It didn't feel like going to a gym, where they're not shouting at you, like, “Go on, do that.” (ID#19)*  *Once it was organised, it was, all very easy to walk in, have the appointment. They called before to remind me of the appointment time. So yeah, any interaction I had with XX community health service was very good. (ID#11)*  *Getting to the program like the reception and stuff was pretty good. (ID#13)*  *One [site] is walking distance from me and the other one I have to drive to. But I choose the one I have to drive to . . . just the whole reception area was much friendlier and easier than the one that I could walk to. So, now I just think, “Well, it’s worth the drive” (ID#3)*  *For months I heard nothing. Nothing was happening with the XX community health service. Nothing. And I thought I was just forgotten type of thing (ID#12)*  *It is no one answers the phone. But you don't even get a person say to you, “Sorry, you're on hold,” you get a record, you know, it keeps going and going. So that's the worst part about it, the contact, to try and contact them [community health service]. (ID#12)*  *Yes, I did have one meeting with [the advanced practice physiotherapist]. I think I was supposed to meet [them], but I didn’t get any reminders and I was actually away. (ID#4)* |
|  |

GLA:D = Good Life osteoArthritis: Denmark
